# Supplementary material for: Effects of Extraction Process Factors on the Composition and Antioxidant Activity of Blackthorn (Prunus spinosa L.) Fruit Extracts
Source: Antioxidants (Basel). 2023 Oct 23;12(10):1897. doi: 10.3390/antiox12101897 (PMC10604850; doi:10.3390/antiox12101897)
Supplement: Supplementary file 1 [file antioxidants-12-01897-s001.zip › antioxidants-2657978-supplementary.pdf]

antioxidants

MDPI

Article

Effects of Extraction Process Factors on the Composition and Antioxidant Activity of Blackthorn (*Prunus spinosa* L.) Fruit Extracts

Ana-Maria Drăghici-Popa [1], Aurelian Cristian Boscornea [2], Ana-Maria Brezoiu [3], Ștefan Theodor Tomas [2], Oana Cristina Pârvulescu [3,*] and Raluca Stan [1,*]

[1] Department of Organic Chemistry, National University of Science and Technology POLITEHNICA Bucharest, 1-7 Gheorghe Polizu St., 011061 Bucharest, Romania; ana_maria.draghici@upb.ro
[2] Department of Bioresources and Polymer Science, National University of Science and Technology POLITEHNICA Bucharest, 1-7 Gheorghe Polizu St., 011061 Bucharest, Romania; cristian.boscornea@upb.ro (A.C.B.); aastomas@hotmail.com (Ș.T.T.)
[3] Department of Chemical and Biochemical Engineering, National University of Science and Technology POLITEHNICA Bucharest, 1-7 Gheorghe Polizu St., 011061 Bucharest, Romania; anamaria.brezoiu@gmail.com
* Correspondence: oana.parvulescu@yahoo.com (O.C.P.); raluca.stan@upb.ro (R.S.)

Supplementary Materials

Table S1. HPLC-PDA characterization of a standard solution mixture.

Compound	*λ* max (nm)	Retention time (min)	Calibration curve	R^2	LOD/LOQ (mg/L)	Linearity domain (mg/L)
Gallic acid	271	3.70	y=8371.14x-1207.10	0.9996	0.11/0.54	0.54-108.00
Protocatechuic acid	279	7.16	y=9007.30x-1186.03	0.9998	0.10/0.49	0.49-98.80
Caftaric acid	326	10.92	y=3741.63x-1004.52	0.9990	0.10/0.50	0.50-100.00
Catechin hydrate	279	12.65	y=1704.55x-1484.97	0.9987	0.52/1.04	0.52-104.00
Chlorogenic acid	326	13.39	y=7634.01x-1686.39	0.9992	0.10/0.49	0.49-99.00
Vanillic acid	292	15.12	y=8457.44x-1216.69	0.9995	0.10/0.49	0.49-98.70
Caffeic acid	323	15.38	y=13188.1x-1501.42	0.9994	0.10/0.49	0.49-99.00
Syringic acid	271	16.80	y=6929.03x-667.27	0.9991	0.11/0.54	0.54-107.30
(-) Epicatechin	279	17.76	y=1585.49x-273.15	0.9993	0.47/0.94	0.94-93.50
Delphinidin chloride	529	21.21	y=13373.70x-5554.22	0.9984	0.11/0.59	0.59-118.00
trans p-Coumaric acid	309	22.17	y=18220.60x-1642.10	0.9972	0.10/0.51	0.51-102.00
Trans-ferulic acid	323	25.46	y=13317.51x-3135.95	0.9997	0.10/0.52	0.52-104.00
Ellagic acid dihydrate	367	25.62	y=5128.27x-577.18	0.9995	0.10/0.51	0.51-71.12
Cyanidin chloride	524	25.67	y=13401.60x-2555.96	0.9990	0.08/0.42	0.42-84.00
Rutin hydrate	355	26.60	y=3813.02x-838.902	0.9992	0.49/0.99	0.99-99.80
Chicoric acid	330	29.18	y=10560.62x-1939.03	0.9996	0.10/0.51	0.51-101.60
Pelargonidin chloride	512	29.64	y=8921.84x-2511.85	0.9993	0.10/0.50	0.50-100.00
Malvidin chloride	535	30.90	y=7243.86x-2363.29	0.9983	0.09/0.46	0.46-92.00
Myricetin	373	31.87	y=9150.32x-1464.36	0.9995	0.10/0.48	0.48-95.00
Rosmarinic acid	330	32.36	y=7282.31x-633.293	0.9988	0.10/0.49	0.49-99.00
trans-Resveratrol	307	33.32	y=17601.10x-2585.25	0.9994	0.100/0.50	0.50-100.00
Quercetin	371	34.87	y=9898.83x-723.173	0.9997	0.10/0.49	0.49-99.00
Kaempferol	367	35.88	y=10549.70x-1296.00	0.9990	0.10/0.48	0.48-97.00

(LOD) limit of detection; (LOQ) limit of quantification.

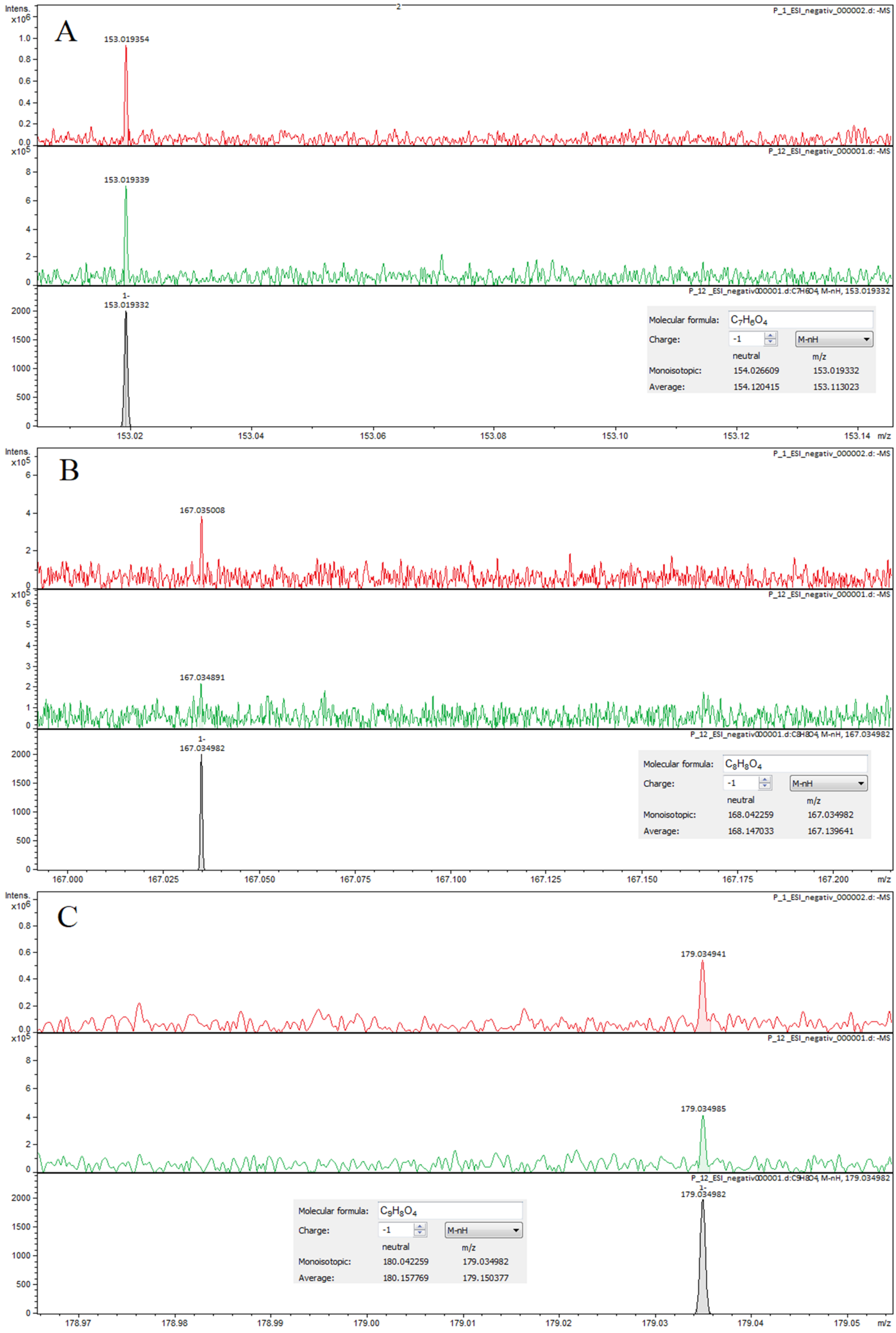
A
B
C
Intens.
153.019354
153.019339
153.019332
P_1_ESI_negativ_000002.d: -MS
P_12 _ESI_negativ_000001.d: -MS
Molecular formula: $C_7H_6O_4$
Charge: -1
M-nH
neutral
m/z
Monoisotopic: 154.026609 153.019332
Average: 154.120415 153.113023
167.035008
167.034891
167.034982
Molecular formula: $C_8H_8O_4$
Monoisotopic: 168.042259 167.034982
Average: 168.147033 167.139641
179.034941
179.034985
179.034982
Molecular formula: $C_9H_8O_4$
Monoisotopic: 180.042259 179.034982
Average: 180.157769 179.150377
m/z

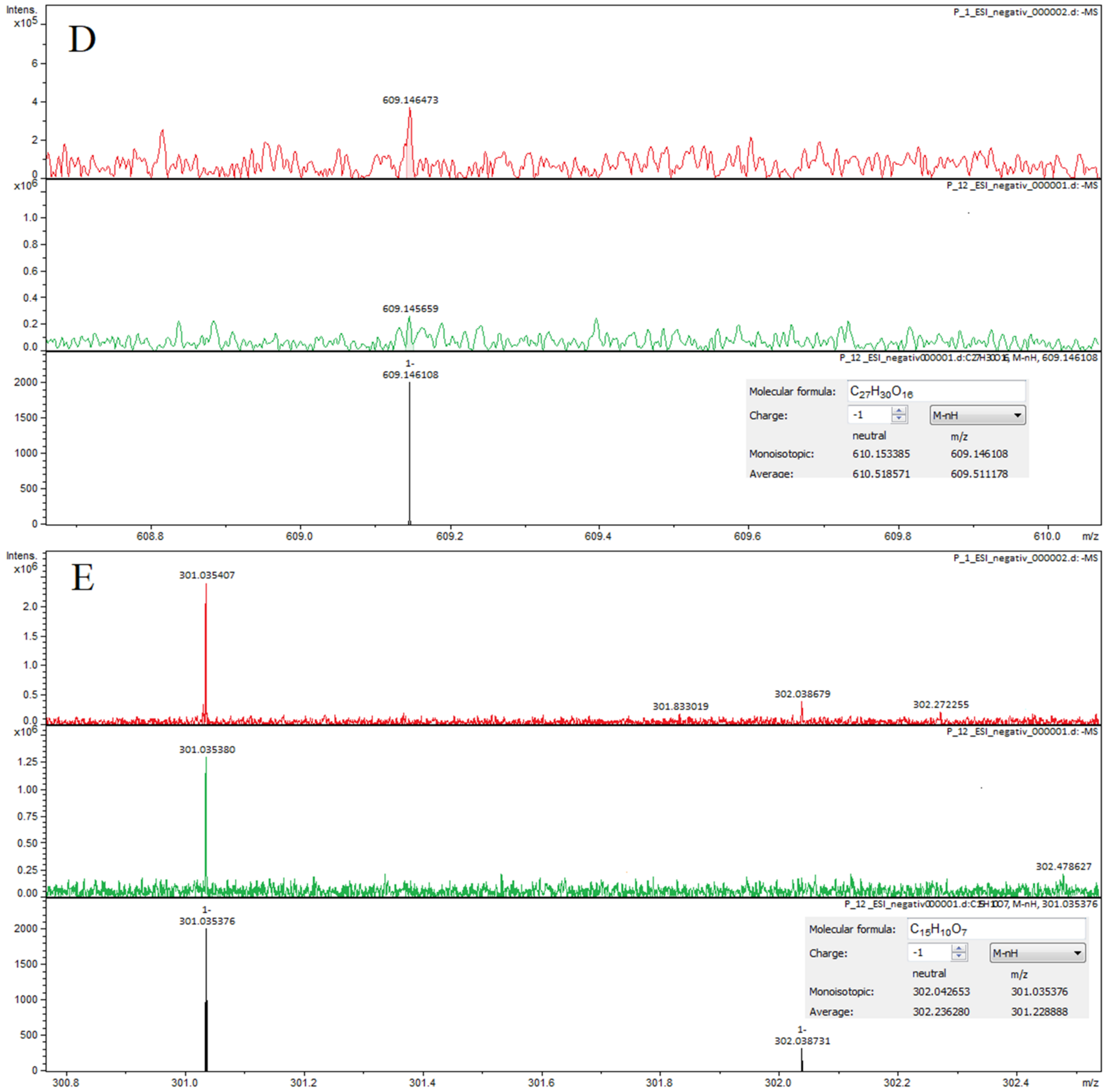

Figure S1. Experimental (red for sample P1 and green for sample P12) and predicted (black) FT-ICR-MS spectra with negative ion mode ESI; (A) protocatechuic acid; (B) vanillic acid; (C) caffeic acid; (D) rutin; (E) quercetin.
